# Visualization of fibroblast activation using ^68^Ga-FAPI PET/CT after pulmonary vein isolation with pulsed field compared with cryoballoon ablation

**DOI:** 10.1007/s12350-023-03220-8

**Published:** 2023-03-21

**Authors:** Jana Kupusovic, Lukas Kessler, Florian Bruns, Jan-Eric Bohnen, Stephan G. Nekolla, Manuel M. Weber, Anna Lauenroth, Manuel Rattka, Ken Hermann, Dobromir Dobrev, Tienush Rassaf, Reza Wakili, Christoph Rischpler, Johannes Siebermair

**Affiliations:** 1 Department of Cardiology and Vascular Medicine, West German Heart and Vascular Center Essen, University of Duisburg-Essen, Hufelandstrasse 55, 45147 Essen, Germany; 2 Department of Nuclear Medicine, University Hospital Essen, University of Duisburg-Essen, Hufelandstrasse 55, 45147 Essen, Germany; 3 Institute of Pharmacology, West German Heart and Vascular Center, University Duisburg-Essen, Essen, Germany; 4 Department of Nuclear Medicine, Klinikum Rechts der Isar, Technical University Munich, Munich, Germany; 5 Department of Molecular Physiology & Biophysics, Baylor College of Medicine, Houston, TX USA; 6 Department of Medicine and Research Center, Montreal Heart Institute and Université de Montréal, Montréal, QC Canada; 7 Department of Nuclear Medicine, Klinikum Stuttgart, Kriegsbergstr. 60, 70174 Stuttgart, Germany; 8 Department of Cardiology and Vascular Medicine, University Hospital Frankfurt, Goethe-University Frankfurt, Theodor-Stern-Kai 7, 60590 Frankfurt am Main, Germany; 9 Krankenhaus Goettlicher Heiland, Dornbacher Strasse. 20-30, 1170 Vienna, Austria

**Keywords:** Atrial fibrillation, pulse field ablation (PFA), pulmonary vein isolation (PVI), fibroblast activation, positron emission tomography (PET), FAPI

## Abstract

**Background:**

Pulsed-field ablation (PFA) is a novel ablation modality for atrial fibrillation (AF) ablating myocardium by electroporation without tissue-heating. With its different mechanism of tissue ablation, it is assumed that lesion creation is divergent to thermal energy sources. ^68^Ga-fibroblast-activation protein inhibitor (FAPI) PET/CT targets FAP-alpha expressed by activated fibroblasts. We aimed to assess ^68^Ga-FAPI uptake in pulmonary veins as surrogate for ablation damage after PFA and cryoballoon ablation (CBA).

**Methods:**

26 patients (15 PFA, 11 CBA) underwent ^68^Ga-FAPI-PET/CT after ablation. Standardized uptake values (SUV) and fibroblast-activation volumes of localized tracer uptake were assessed.

**Results:**

Patient characteristics were comparable between groups. In PFA, focal FAPI uptake was only observed in 3/15 (20%) patients, whereas in the CBA cohort, 10/11 (90.9%) patients showed atrial visual uptake. We observed lower values of SUV_max_ (2.85 ± 0.56 vs 4.71 ± 2.06, *P* = 0.025) and FAV (1.13 ± 0.84 cm^3^ vs 3.91 ± 2.74 cm^3^, *P* = 0.014) along with a trend towards lower SUV_peak_ and SUV_mean_ in PFA vs CBA patients, respectively.

**Conclusion:**

Tissue response with respect to fibroblast activation seems to be less pronounced in PFA compared to established thermal ablation systems. This functional assessment might contribute to a better understanding of lesion formation in thermal and PFA ablation potentially contributing to better safety outcomes.

**Supplementary Information:**

The online version contains supplementary material available at 10.1007/s12350-023-03220-8.

## Introduction

Pulmonary vein isolation (PVI) is the interventional cornerstone therapy for atrial fibrillation (AF).^1^ Cryoballoon ablation (CBA) has become an established single-shot-ablation technique achieving PVI by circumferential ablation lesions.^2^ Major complications such as atrio-esophageal fistula or phrenic nerve palsy after thermal ablation are rare but still of concern.^3^ Introduction of pulse field ablation (PFA) as a novel single-shot modality promises an equally effective, yet safer modality due to its tissue specificity and consequently absence of significant collateral damage to nearby structures (particularly the esophagus, vessels and phrenic nerve).^4,5^ PFA employs trains of short-duration high amplitude electrical pulses that specifically ablate cardiomyocytes within the myocardial tissue by forming irreversible nanoscale pores leading to apoptosis through electroporation without tissue heating.^5^ With its different mechanism of tissue ablation, it is assumed that tissue damage and consecutive lesion creation is divergent to thermal energy sources.^6,7^

**Figure 1:**
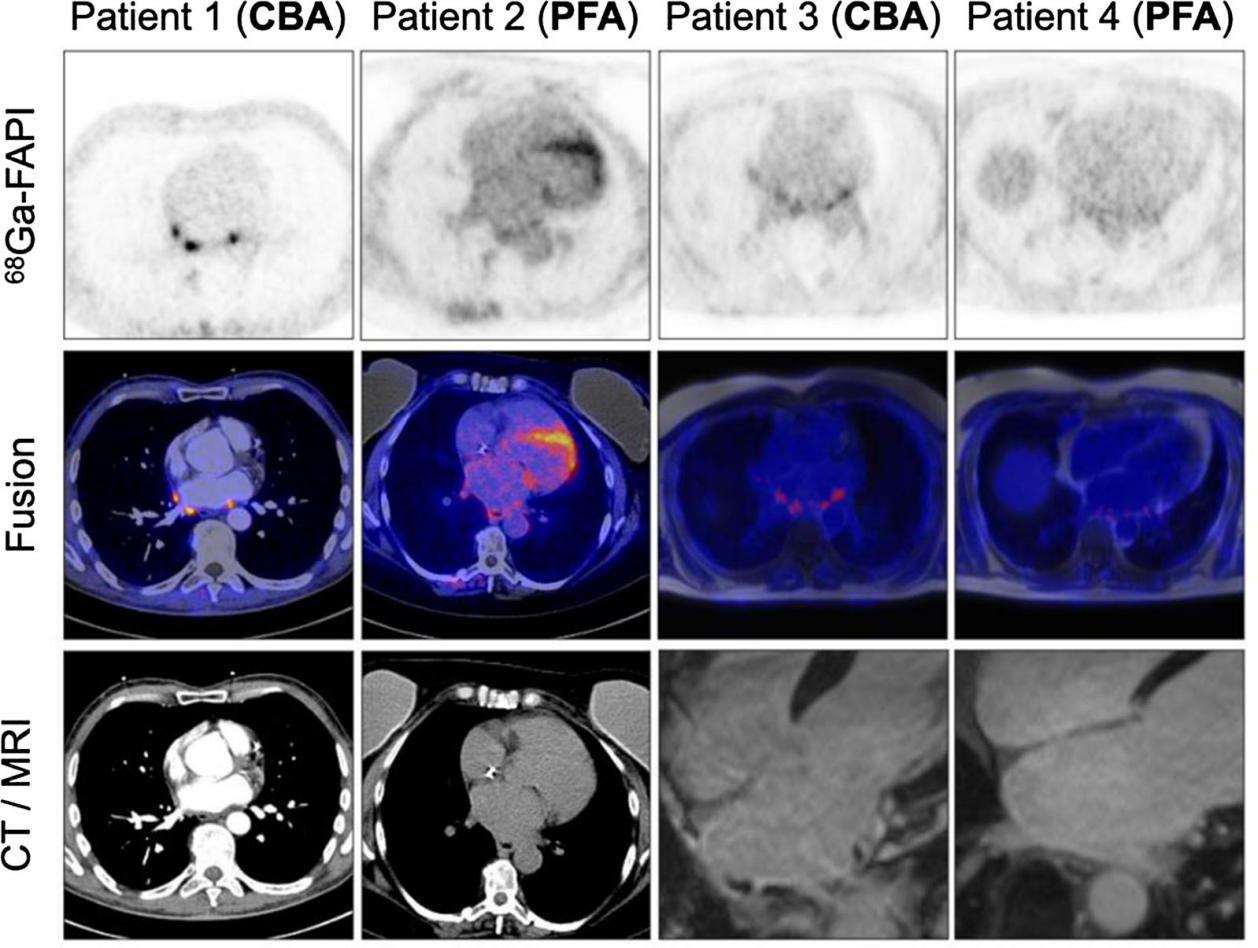
Patient 1 received CBA and showed intense tracer uptake in PVs; Patient 2 underwent PFA 7 days before imaging and showed mildly increased tracer uptake in the ostia of PVs. Of note, this patient had persistent AF, as well as severely dilated left atrium (left atrial volume of 40 mL·cm^−2^) which might have contributed to FAPI uptake as well. Incidental diffuse uptake in the left ventricle may possibly reflect myocardial remodeling in the setting of DCM; Patient 3 having undergone CBA and PET/MRI revealed moderate tracer uptake with corresponding late gadolinium enhancement (LGE) of the left atrium; Patient 4: received PFA and no visual tracer uptake can be observed in PET nor LGE in MRI. *CBA*, cryoballoon ablation; *PVs*, pulmonary veins; *PFA*, pulse field ablation; *PET*, positron emission tomography; *CT*, computer tomography; *MRI*, magnetic resonance imaging

We have already shown the feasibility of hybrid imaging using ^68^Ga-Fibroblast-activation protein inhibitor (FAPI) positron-emission tomography (PET) in assessing thermal damage in post PVI patients (cryo and radiofrequency energy) compared to controls.^8^ As PFA uses non thermal energy to induce cell death, the aim of our study was to assess FAPI uptake in patients after PFA as a surrogate for ablation damage and compare it with uptake after CBA. The hypothesis was that patients treated with PFA will have a different extent of tracer uptake due to its selective mechanism of irreversible electroporation resulting in apoptosis of cardiomyocytes opposed to CBA-induced nonselective tissue necrosis with collateral microvascular obstruction or intramural hemorrhage (Figure 1).

## Methods

### Patient population

Twenty-six patients who had undergone ^68^Ga-FAPI-PET after PVI were included. Five patients matched for age, left ventricular ejection fraction without a history of AF or cardiac ablation having undergone ^68^Ga-FAPI-PET for tumor staging served as controls. Patients treated with cardio-toxic drugs like anthracyclines or immune checkpoint inhibitors, or chest radiation were excluded to avoid distortion of myocardial FAPI uptake due to confounders.^9^ We assessed clinical baseline characteristics, imaging parameters from echocardiography and procedural PVI data as well as 6 months follow-up data. The investigations were conducted in accordance with the Declaration of Helsinki and national regulations. All patients gave written informed consent to undergo ^68^Ga-FAPI PET/computer tomography (CT) or ^68^Ga-FAPI PET/magnetic resonance (MRI) following the regulations of the German Pharmaceuticals Act §13(2b). Retrospective analysis of PET hybrid imaging and clinical data was approved by the local ethics committee for the purpose of the present study (Permit No. 20-9777-BO).

### Catheter ablation

All procedures were performed under uninterrupted oral anticoagulation and under continuous heparin administration during the procedure guided by activated clotting time goal of 300-350 ms. All PVI procedures were performed under 3D high-density mapping guidance (Rhythmia HDx™, Boston Scientific, Massachusetts, United States) with a diagnostic coronary sinus catheter, an additional catheter in right ventricle or right atrium (RA), respectively for phrenic nerve capture if required. Transseptal puncture and 3D mapping were performed as previously described.^10^

In all patients, the procedural endpoint was complete PVI. In the PFA group, a 12-French over-the-wire PFA ablation catheter (Farawave, Farapulse, Inc., California, United States) was used in either a flower or olive configuration delivering the energy in a set of microsecond-scale biphasic pulses of 1800-2000 Volts in bipolar approach across all electrodes. Each application was made of five pulse packets delivered over a few seconds. Applications were repeated eight times per vein, 4 times in flower, 4 times in olive configuration, with repositioning and/or rotation of the catheter every two applications to ensure circumferential PV ostial and antral coverage. The procedural endpoint of complete PVI was confirmed by high-density mapping after the PFA applications.

In patients undergoing CBA, PVI was performed using a 28-mm cryoballoon catheter (POLARx™, Boston Scientific, Massachusetts, United States) under fluoroscopic guidance. The occlusion of PV was confirmed with the retention/leakage of the contrast agent after injection at the balloon’s distal tip. A minimum of two freezes was delivered to each PV with a targeted duration of 240 seconds. After PVI, the entrance block was confirmed by placing an electrode catheter (Polar map catheter) within the PVs, and the exit block by pacing from the catheter.

Follow-up care after an ablation included physical examination and 24 hours Holter-Monitoring in 3 and 6 month intervals post PVI. AF recurrence was defined as any documented AF or flutter > 30 seconds after blanking period of 3 months.

### Radiotracer synthesis

The radiotracer ^68^Ga-FAPI-46, a recently developed FAPI compound, has substantially improved ratios of tumor-to-blood, liver, muscle, and intestinal uptake compared to other FAPI radiotracers. The synthesis of ^68^Ga-FAPI-46 was performed as previously described.^11^

### Image acquisition

PET scans were performed on a PET/CT system (Biograph Vision, Siemens Healthineers, Erlangen, Germany). Injected activity of ^68^Ga-FAPI was 113.5 ± 27.8 MBq [73;165 MBq]. ECG-gated cardiac PET imaging was performed approximately 20 minutes p.i. Low-dose CT was performed for attenuation correction (30 mAs, 120 keV, 512 × 512 matrix, 3 mm slice thickness). In addition, a CT angiography (CTA) was performed (30-40 mL iomeprol; 400 mg iodine per milliliter; Iomeron 400; Bracco, Milan, Italy) with the following parameters: spiral mode, 0.6 seconds gantry rotation; collimation, 64 × 0.6 mm; pitch, 1.375:1; section thickness, 0.6 mm; reconstruction interval, 0.5 mm; tube voltage: 120 kV; current intensity: 300 mA. Three patients underwent PET/MRI (Biograph mMR, Siemens Healthcare, Erlangen, Germany) with PET acquisition in 1 bed position and 3D image reconstruction (2 × 2 × 2 mm voxel size) using ordinary Poisson ordered subset expectation maximization with 3 iterations and 21 subsets, a Gaussian filter with 5.0 mm full width at half-maximum and a 344 × 344 image matrix. For automatic attenuation correction of the acquired PET data, a four-compartment model attenuation map was calculated from fat-only and water-only Dixon-based sequences by segmentation into background, lung, fat, and soft tissue.

### Image evaluation

PET data was analyzed by two nuclear medicine specialists (CR and LK) on a consensus decision. Tracer uptake was visually rated as “intense”, “moderate” or “absent” if uptake was clearly higher, slightly higher or comparable to blood pool in RA, respectively. Tracer uptake was quantified as maximum (SUV_max_), peak (SUV_peak_) and mean (SUV_mean_) standardized uptake values (SUV) from static images 20 minutes after tracer injection. For this purpose, a region grow algorithm at the PV ostia with patient individual threshold of mean uptake in the RA + 2 standard deviations (Syngo.via software; Siemens Healthineers, Erlangen, Germany) and fibroblast activation volume-of-interest (FAV) for each PV ostium was defined. Background (bloodpool, RA) was quantified using a circular 1 cm diameter sphere.

### Statistical analysis

Statistical analysis was performed using SPSS statistics and GraphPad Prism (version 8.4.2; GraphPad Software, San Diego, California USA), with quantitative values expressed as mean ± standard deviation or median and range where appropriate. Comparison of non-parametric data was performed using a Mann–Whitney-*U*-test or Kruskal–Wallis test for multiple comparisons. All tests were performed two-sided and a *P* value < 0.05 was considered to indicate statistical significance.

## Results

### Patient characteristics and follow-up regarding AF recurrence

A total of n = 26 patients (CBA = 11, PFA = 15) were retrospectively analyzed with respect to the tracer uptake in pulmonary veins after PVI. Of those n = 7 CBA have been previously reported.^8^ Five ablation-naïve patients without AF, matched for age, left ventricular ejection fraction (LVEF), who were scanned as a part of their oncological follow-up served as controls.^8^ All 11/11 CBA patients, 66.7% (10/15) of PFA patients and 80% (4/5) of controls were male. There was no statistical significance in difference of proportion of patients with paroxysmal AF between CBA (36.4%) and PFA (60.0%). The overall baseline characteristics of the three study groups were balanced for age, LVEF, left atrial volume index, arterial hypertension and chronic heart failure. Only one case of AF recurrence in the CBA cohort was registered in 6 months follow-up compared to n = 0 cases in the PFA cohort. Data on patient characteristics is provided in Table 1.

**Table 1:** Patient characteristics

Procedure	CBA, n = 11	PFA, n = 15	Controls, n = 5	*P*
Age at scan, years	65.1 ± 9.4	65.3 ± 10.2	58.4 ± 3.8	0.3
Male sex, n (%)	11 (100)	10 (66.7)	4 (80)	0.1
BMI, kg·m^−2^	27.9 ± 4.5	28.8 ± 4.9	25.8 ± 4.6	0.6
Paroxysmal AF, n (%)	4 (36.4)	9 (60.0)	N/A	0.4
LVEF, %	55.2 ± 5.7	52.7 ± 8.8	57.2 ± 7.6	0.5
CHF, n (%)	5 (45.5)	7 (46.7)	1 (20)	0.6
LAVI, mL·cm^−2^	38.7 ± 18.4	34.3 ± 9.4	25.2 ± 3.4	0.2
History of CAD, n (%)	4 (36.4)	5 (33.3)	0 (0)	0.3
Cardiovascular risk factors				
Hyperlipoproteinemia, n (%)	5 (45.5)	9 (60.0)	1 (20)	0.3
Arterial hypertension, n (%)	10 (90.9)	12 (80.0)	3 (60)	0.4
Diabetes, n (%)	1 (9.1)	2 (13.3)	1 (20)	0.9
Prior Stroke, n (%)	0 (0)	2 (13.3)	0 (0)	0.3
CHA_2_DS_2_ VASc score	2.4 ± 1.5	2.6 ± 1.2	N/A	0.7
Time to FAPI, days (median)	24.9 ± 14.3 (21)	22.9 ± 13.4 (20)	N/A	0.7
Creatinine, mg·dL^−1^	1.1 ± 0.2	1.1 ± 0.3	0.8 ± 0.2	0.2

*CBA*, cryoballoon ablation; *PFA*, pulse field ablation; *BMI*, body mass index; *AF*, atrial fibrillation; *N/A*, not applicable; *LVEF*, left ventricular ejection fraction; *CHF*, chronic heart failure; *LAVI*, left atrial volume index; *CAD*, coronary artery disease

### Procedural characteristics

Fluoroscopy time was significantly higher in the PFA group (PFA = 31.1 ± 9.8 minutes vs CBA = 23.1 ± 7.0 minutes, *P* = 0.03), while amount of administered contrast agent was higher in CBA cohort (CBA = 167.5 ± 84.4 mL vs PFA = 104.7 ± 40.8 mL, *P* = 0.01). Total procedural time, incl. 3D mapping before and after ablation was balanced between the two groups (CBA: 177.3.0 ± 56.5 minutes vs PFA: 179.3 ± 49.3 minutes, *P* = 0.92). Other procedural data are listed in Table 2.

**Table 2:** Procedural data

Procedural data	CBA	PFA	p
Radiation dose, cGy	2024.4 ± 1413.5	2574.1 ± 1668.0	0.37
Fluoroscopy time, minutes	23.1 ± 7.0	31.1 ± 9.8	0.03
Total procedure time, minutes	177.3.0 ± 56.5	179.3 ± 49.3	0.92
Administered contrast, mL	167.5 ± 84.4	104.7 ± 40.8	0.01
CBA			
Number of freezes, n	7.6 ± 2.0		
Max. temperature, °C	− 61.1 ± 5.1		
Total freeze time, seconds	1216.0 ± 427.6		
PFA			
Number of impulses			
Flower configuration, n	16 ± 0		
Olive configuration, n	16 ± 0		

### Visual and quantitative tracer uptake after PFA vs CBA procedures

In the PFA group only 3/15 (20%) patients showed visual uptake (intense), whereas 80% of PFA patients showed no visual tracer uptake at all. In the CBA cohort, 9/11 (81.8%) patients showed intense, 1/11 (9.1%) moderate visual uptake in PV ostia, while no uptake was observed in only 1/11 patient.

**Figure 2:**
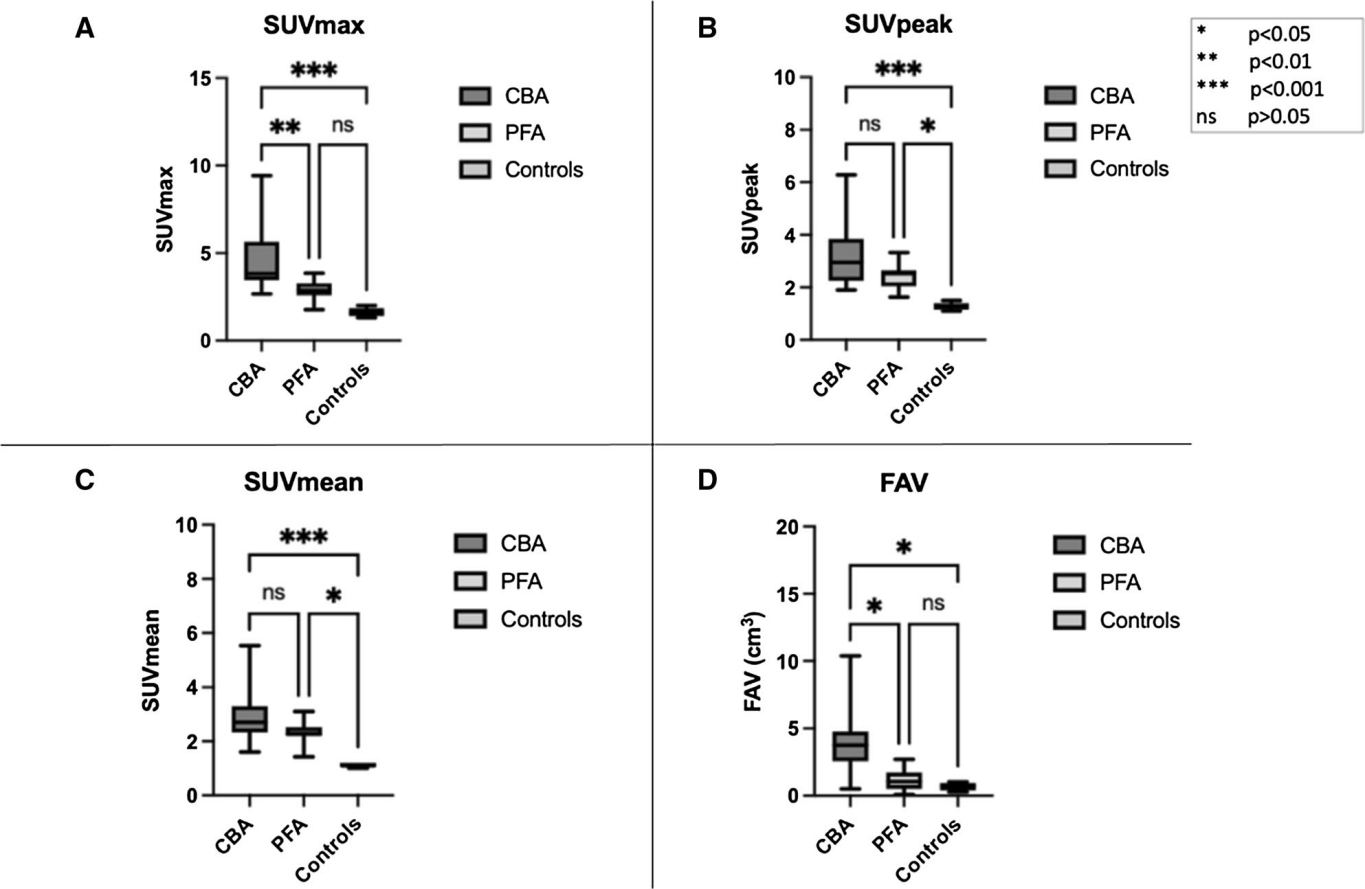
The distribution of the specific PET parameters SUV_max_ (**A**), SUV_peak_ (**B**), SUV_mean_ (**C**) and FAV (**D**) between uptake at PV antra in CBA, PFA and ablation-naïve individuals (controls), demonstrating quantitatively significantly lower tracer uptake in PFA patients in comparison to CBA for SUV_max_ (**A**) and FAV (**D**) with a clear, albeit non-significant lower values of SUV_peak_ (**B**) and SUV_mean_ (**C**) for PFA. All PET parameters after CBA were significantly higher than in controls (**A-D**). PFA patients had significantly higher tracer uptake in comparison to controls for SUV_peak_ (**B**) and SUV_mean_ (**C**) with a trend of higher uptake for SUV_max_ (**A**) and FAV (**D**) *CBA*, cryoballoon ablation; *PFA*, pulse field ablation *SUV*, standardized uptake value; *FAV*, fibroblast activation volume

We observed significantly lower values of SUV_max_ (2.85 ± 0.56 vs 4.71 ± 2.06, *P* = 0.025) and FAV (1.13 ± 0.84 cm^3^ vs 3.91 ± 2.74 cm^3^, *P* = 0.014) in PFA vs CBA patients, respectively. Further, there was a strong trend towards lower SUV_peak_ (2.43 ± 0.42 vs 3.24 ± 1.25, *P* = 0.25) and SUV_mean_ (2.30 ± 0.46 vs 2.87 ± 1.10, *P* = 0.45) for PFA compared to CBA patients, respectively. A significantly higher uptake in CBA procedures compared to controls could be shown for all PET parameters. Patients after PFA showed a trend of higher uptake than controls, reaching the boundaries of statistical significance for SUV_peak_ (2.43 ± 0.42 vs 1.28 ± 0.15 *P* = 0.02) and SUV_mean_ (2.30 ± 0.46 vs 1.12 ± 0.08 *P* = 0.014). Figure 2 exemplarily depicts the tracer uptake in the study groups.

### Temporal changes of FAPI uptake after PFA and CBA ablation

**Figure 3:**
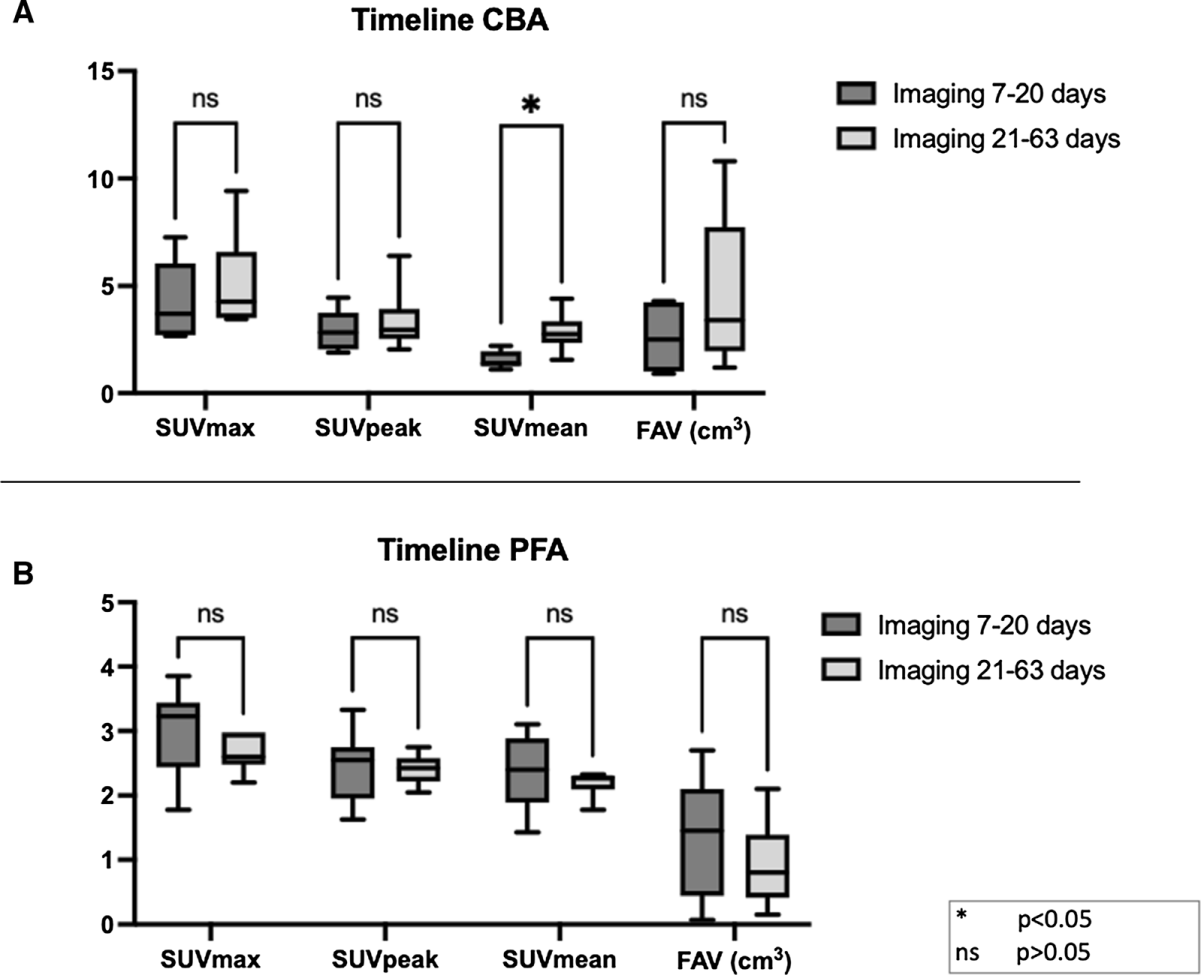
PET parameters after CBA and PFA stratified for time interval between ablation and FAPI scan: (**A**) Figure showing a clear tendency of lower uptake of PET parameters in CBA patients that were imaged within first 20 days of CBA ablation, with SUV_mean_ reaching the boundaries of significance. (**B**) In PFA patients, there was trend of higher uptake of SUV_max_, SUV_peak_, SUV_mean_ and FAV within the first 20 days of ablation with PFA without reaching statistical significance. *CBA*, cryoballoon ablation; *PFA*, pulse field ablation; *SUV*, standardized uptake value; *FAV*, fibroblast activation volume

Further, we sought to evaluate a potential temporal change of FAPI tracer uptake after both single-shot modalities. For this purpose, we stratified both cohorts according to the time of imaging (Figure 3), differentiating between patients that have been scanned within first 20 days after PVI (9/15 and 5/11 patients of PFA and CBA cohorts) and patients that were scanned 21-63 days post ablation. In PFA patients, we observed a trend of higher FAPI uptake in all PET parameters within the early phase (< 20 days) post ablation (SUV_max_: 2.97 ± 0.67 vs 2.65 ± 0.29, *P* = 0.21; FAV: 1.28 ± 0.93 vs 0.92 ± 0.69, *P* = 0.46). However, none of the PET parameters reached the boundaries of statistical significance.

The opposite held true in the CBA cohort, where a trend of lower FAPI uptake was observed within the early phase (SUV_max_: 4.24 ± 1.90 vs 5.09 ± 2.28, *P* = 0.54; SUV_peak_: 2.89 ± 0.99 vs 3.36 ± 1.54, *P* = 0.66; FAV: 2.60 ± 1.62 vs 4.62 ± 3.56, *P* = 0.43) with SUV_mean_ reaching the boundaries of significance (1.56 ± 0.42 vs 2.84 ± 0.91, *P* = 0.02).

## Discussion

This retrospective study is the first comparing FAPI uptake as surrogate for fibroblast activation in patients after single shot non-thermal ablation with PFA to thermal ablation with CBA. Patients treated with PFA had significantly lower FAPI uptake compared to individuals treated with CBA indicating less pronounced fibroblast activation after non-thermal ablation by electroporation.

### Tracer uptake after nonthermal vs thermal ablation

Our study found that visual tracer uptake was significantly lower in patients after PFA compared to CBA procedures, as only 3/15 (20%) of PFA patients had a positive visual uptake compared to 10/11 (90.9%) CBA patients who had intense-to moderate visual uptake. These results were in line with quantitative analyses showing significantly lower quantitative uptake in PVs after PFA in comparison to CBA-treated individuals.

The significantly lower extent of tracer uptake as a surrogate for a lower degree of fibrotic remodeling after PFA in comparison to CBA could be explained through different mechanism of energy delivery. PFA, using a predefined protocol, causes selective electroporation of cardiomyocytes leading to apoptosis, thereby sparing the surrounding vascularized connective tissue, which seems to be less sensitive for the used set-up of electroporation.^4,12,13^ In contrast, CBA results in a non-selective coagulatory necrosis of both the target area and the surrounding tissue, affecting both cardiomyocytes as well as nearby microvascular structures which may translate in different magnitude/pathways of inflammatory vs fibrotic reactive processes in terms of tissue remodeling/healing.^14,15^ As PFA has only recently been introduced published data in regard to imaging is scarce. A recent MRI study of patients after ablation with both non-thermal and thermal modalities identified a large area of late-gadolinium enhancement (LGE) in PFA patients directly after ablation, which, however, almost disappeared in the 3-months follow up scan.^16^ This was in complete contrast to an initially small non-homogenous LGE after CBA which then persisted three months later.^16^ The authors suggested that the initial (extensive) LGE after PFA was induced by acute disintegration of the sarcolemma, resulting in tissue oedema. The regredient LGE after 3 months was explained by the lack of development of chronic fibrosis.^16^ The chronic disappearance of LGE after PFA is an interesting finding and in accordance with our results as we observed significantly lower amount of FAPI uptake in PFA patients compared to CBA within the first weeks post PVI suggesting a lower degree of activation of fibroblasts in PFA and in consequence lack of fibrosis or absence of LGE as a surrogate of fibrosis. We hypothesize that cardiomyocyte apoptosis after PFA, opposed to general tissue necrosis after CBA, does not trigger an inflammatory cascade that ultimately causes fibroblast activation by the transformation from inflammation to fibrosis by stimulating collagen synthesis.^16–18^ In contrast, CBA, as a thermal modality causing coagulatory necrosis, is suggested to trigger an inflammatory response, hence activating fibroblasts that may be responsible for LGE persistence as well as significantly increased FAPI uptake in these patients.^14,16^ Further, the disruption of structural integrity as a consequence of thermal ablation due to microvascular obstruction or intramural hemorrhage may lead to mechanical strain on fibroblasts, stimulating their activation, while after an ablation with PFA the structural integrity of extracellular matrix is preserved, preventing the additional mechanical stress on fibroblasts.^7,14,16,19^

### Fibroblast activation after thermal/non-thermal ablation vs controls

The finding of significantly higher fibroblast activation after CBA, which seems to further increase over time, has been reported and discussed elsewhere.^8^ Here we report a positive visual uptake in 3/15 (20%) of PFA patients and the finding that FAPI uptake in PFA patients tended to be higher compared to ablation-naïve controls in SUV_mean_ and SUV_peak_ parameters. This finding suggests divergent extent of fibroblast activation in patients after PVI with a clear increase after CBA and a less pronounced, but still to some degree enhanced level of fibroblast activation after PFA. At the moment one can only speculate about this finding. Preclinical studies have shown that although apoptosis is the dominant pathway of cell death in PFA, some degree of immediate cell necrosis after PFA energy delivery may be present, possibly triggering a low-level inflammation cascade that further results in activation of fibroblasts.^5,18,20^ Another reason for this finding could be found in the direct contact of the PFA device. Especially the olive configuration where the PV ostium is deeply intubated with the PFA device might lead to the triggering of a local fibroblast activation by mechanical stimulation as a consequence of a direct myocardial contact.^21,22^

Last, the increased FAPI uptake in PFA vs controls could be suggested in the pathophysiologic characteristics of the AF itself, as chronic inflammation and increased fibrotic atrial burden have been increasingly recognized as an important pathomechanism for AF in terms of an atrial cardiomyopathy and no serial pre-ablation imaging was performed in the PFA patients to determine the pre-ablation/baseline FAPI status in these patients.^23–27^ Both chronic atrial inflammation as well as atrial fibrosis may explain the increased level of FAPI uptake in PFA patients, opposed to non-AF controls.

As an incidental finding, a diffuse left ventricular FAPI uptake was observed in one PFA patient with dilated cardiomyopathy (DCM) and reduced LVEF, possibly reflecting ongoing cardiac remodeling in the setting of DCM, which is further highlighted by the diffuse pattern of ventricular uptake.^28^ This would be in line with reports of previous studies which associated decreased LVEF with FAPI uptake as well as recent preclinical study, where in rats with heart failure a positive FAPI uptake was observed as a result of an ongoing myocardial fibrosis development.^9,11,29^

### Temporal relation of FAPI uptake depending on the imaging timepoint after PFA/CBA

The MRI study on PFA patients observed LGE disappearance after 3 months post PFA.^16^ Analogous to this study we tried to evaluate if there is a trend of lower FAPI uptake after PFA procedures over time. After stratifying the PFA cohort according to the time of the imaging we observed only a trend of higher FAPI uptake within the early phase without significant changes in tracer uptake between the time points of imaging. However, preclinical data do suggest that activation of fibroblasts happens within the first 28 days following myocardial injury due to myocardial infarction,^30^ and published preclinical data suggest peak FAPI uptake to be 6 days after myocardial injury.^31^ Although the results of our study might have been hampered by the small patient cohort and/or the overall low uptake, due to the specific tissue response following PFA, there might be a trend of decreasing fibroblast activation after PFA. On the other side in CBA patients there was a clear increase after PVI and even a sign of further increase in FAPI uptake over time in the late vs early CBA cohort further underlying a different effect of the ablation technologies on the cellular level.

### Clinical implication and future perspectives

Despite the lack of chronic LGE or fibroblast activation, the follow-up data of PFA studies and the results of our 6-month follow-up suggest that recurrence rate is not inferior to thermal ablation.^6,16^ This is also underlined by the first reported 1-year outcome data of PFA compared to thermal PVI.^6^ This is an encouraging finding suggesting that PFA is simultaneously a noninferior modality with respect to AF recurrence while simultaneously being a safer modality with respect to adverse effects of increased fibrosis and its complications observed after thermal ablation including PV stenosis, substrate for macro-reentry arrhythmias or atrial stiffness due to adverse remodeling.^5,6,32^

Protocols of PFA utilization regarding the use of either monophasic or biphasic waveforms have been modified according to the increasing experience and while results of PFA have been promising in regard to safety and outcomes, we still don´t know its possibilities in regard to overpowering.^5^ Imaging with FAPI may offer a chance to monitor different PFA protocols in regard to their configuration through level of FAPI uptake as surrogate for fibroblast activation. Further, FAPI imaging, especially in combination with LGE-MRI and functional follow-up parameters of myocardial function may allow to better understand subsequent myocardial remodeling due to both thermal and non-thermal ablation caused lesions as well as mechanisms behind AF recurrence. The question if there could be a direct effect of the electroporation on the fibroblast activity, which could in consequence alter the progress of atrial fibrosis, remains completely speculative and has to be elucidated by future studies.

### Limitations

This was an observational study of small cohorts with no histological validation, nor a pre-ablation imaging in the ablation patients. Larger prospective studies with repeat hybrid imaging with PET and LGE-MRI in combination with electrophysiological studies, histological validation, longer follow-up and cardiac biomarkers as well as concentration of fibroblast activation protein in serum are needed to further explore the significance of this tracer in evaluation of structural changes following different PVI modalities.

## New Knowledge Gained

Tissue response with respect to fibroblast activation seems to be less pronounced in non-thermal single shot ablation system with PFA compared to established thermal single shot ablation systems.

Timeline of peak fibroblast activation seems to occur earlier in a non-thermal ablation with PFA, compared to a thermal ablation with CBA.

## Conclusion

Ablation with PFA led to lower levels of fibroblast activation compared to thermal ablation with CBA, reflecting different cell death mechanism and collateral remodeling processes induced by these ablation techniques. Since PFA is a relatively novel ablation modality, imaging with ^68^Ga-FAPI-PET may help to understand lesion formation after PFA and how it relates to a long-term outcome or possible complications, that at the moment may not be apparent. ^68^Ga-FAPI-PET may be used as an imaging modality to monitor atrial remodeling in response to tissue damage.

### Supplementary Information

Below is the link to the electronic supplementary material.

Supplementary file1 (PDF 479 KB)

Supplementary file2 (MP3 4495 KB)

## Data Availability

Data available on request from the authors upon reasonable request.

## Abbreviations

PVI: Pulmonary vein isolation
AF: Atrial fibrillation
CBA: Cryoballoon ablation
PFA: Pulse field abaltion
PET/CT: Positron emission tomography/computed tomography
FAPI: ^68^ Ga-Fibroblast-activation protein inhibitor
MRI: Magnetic resonance imaging
LVEF: Left ventricular ejection fraction
LGE: Late gadolinium enhancement
SUV: Standardized uptake value
