# Supplementary material for: Visualization of fibroblast activation using 68Ga-FAPI PET/CT after pulmonary vein isolation with pulsed field compared with cryoballoon ablation
Source: J Nucl Cardiol. 2023 Mar 21;30(5):2018–28. doi: 10.1007/s12350-023-03220-8 (PMC10558367; doi:10.1007/s12350-023-03220-8)
Supplement: Supplementary file 1 — Supplementary file1 (PDF 479 KB) [file 12350_2023_3220_MOESM1_ESM.pdf]

Journal of Nuclear Cardiology | Official Journal of the American Society of Nuclear Cardiology

Visualization of fibroblast activation using ^{68}Ga-FAPI PET/CT after pulmonary vein isolation with pulsed field compared with cryoballoon ablation

J. Kupusovic[1]*, L. Kessler[2]*, F. Bruns[1,3], J. Bohnen[1], S. Nekolla[4], M. Weber[2], A. Lauenroth[2], M. Rattka[1], K. Hermann[2], D. Dobrev[3,5,6], T. Rassaf[1], R. Wakili[1]*, C. Rischpler[2]*, J. Siebermair[1]*

[1] Department of Cardiology and Vascular Medicine, University of Duisburg-Essen, Hufelandstrasse 55, 45147 Essen, Germany

[2] Department of Nuclear Medicine, University Hospital Essen, University of Duisburg-Essen, Hufelandstrasse 55, 45147 Essen, Germany

[3] Institute of Pharmacology, West German Heart and Vascular Center, University Duisburg-Essen, Essen, Germany

[4] Department of Nuclear Medicine, Klinikum Rechts der Isar, Technical University Munich, Munich, Germany

[5] Department of Molecular Physiology & Biophysics, Baylor College of Medicine, Houston, Texas, United States of America

[6] Department of Medicine and Research Center, Montreal Heart Institute and Université de Montréal, Montréal, Quebec, Canada

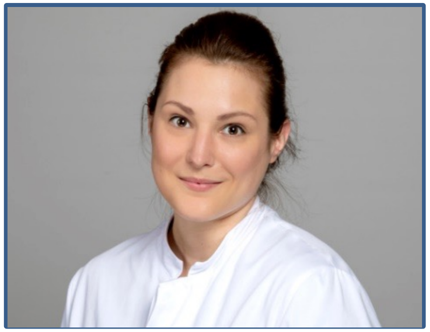

DAS HERZ IN ESSEN

Copyright American Society of Nuclear Cardiology

ASNC Springer

BACKGROUND

1- Pulsed-field ablation (PFA) is a novel ablation modality for atrial fibrillation (AF) ablating myocardium by electroporation without tissue-heating.

2- With its different mechanism of tissue ablation, it is assumed that lesion creation is divergent to thermal energy sources.

3- ^{68}Ga-fibroblast-activation protein inhibitor (FAPI) PET/CT targets FAP-alpha expressed by activated fibroblasts.

4- Aim: To assess ^{68}Ga-FAPI uptake in pulmonary veins as surrogate for ablation damage after PFA and cryoballoon ablation (CBA).

Copyright American Society of Nuclear Cardiology

ASNC Springer

METHODS

A. Study type: Retrospective

B. Study subjects: 26 patients who had undergone ^{68}Ga-FAPI-PET after pulmonary vein isolation with CBA (n=11) or PFA (n=15) as well as controls (n=5) matched for age, LVEF without a history of AF or cardiac ablation

C. Study endpoints:To assess and compare visual and quantitative FAPI uptake as a surrogate for ablation damage in patients after PFA and CBA with hypothesis that patients treated with PFA will have a different extent of tracer uptake due to its selective mechanism of cell death opposed to cryoablation-induced nonselective tissue necrosis

D. Study variables: Independent: Type of Ablation (CBA vs. PFA vs. non-ablated controls), Dependent: PET Uptake parameters

Copyright American Society of Nuclear Cardiology

RESULTS

Examples of visual ^{68}Ga-FAPI PET/CT uptake after CBA and PFA:

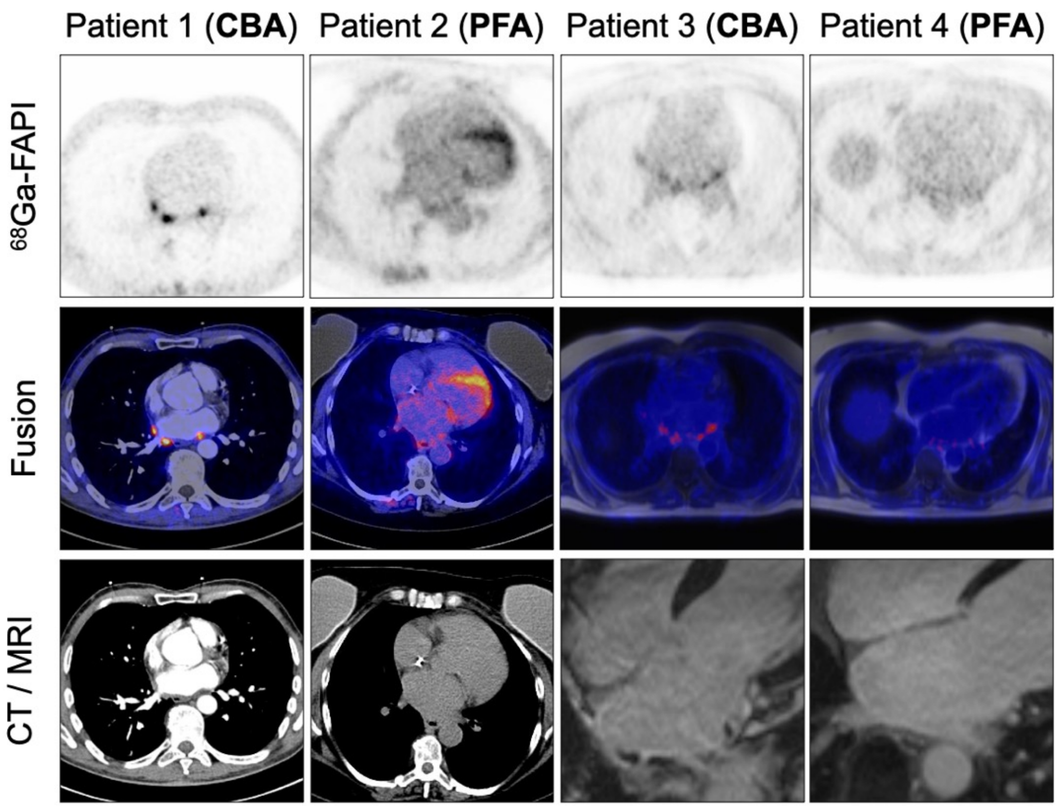

- Patient 1 (CBA): intense tracer uptake in pulmonary veins (PV) after CBA
- Patient 2 (PFA): mildly increased tracer uptake in ostia of PVs after PFA.
- Patient 3 (CBA:) moderate tracer uptake with corresponding late gadolinium enhancement (LGE) after CBA
- Patient 4 (PFA): no tracer uptake nor LGE after PFA

Copyright American Society of Nuclear Cardiology

RESULTS

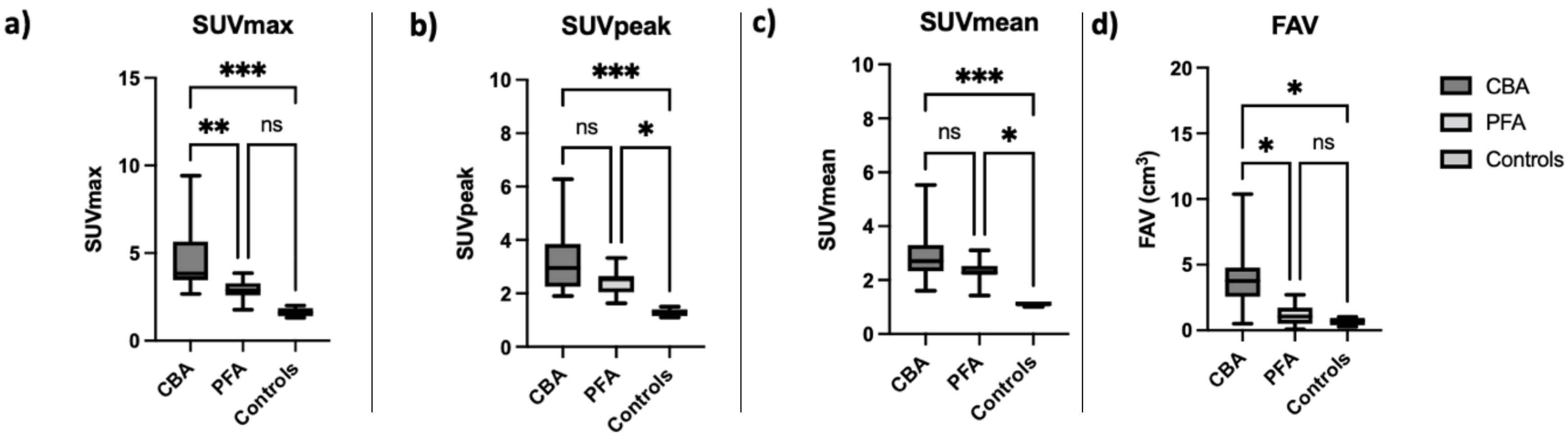

The distribution of the specific PET parameters between uptake at PV antra in CBA, PFA and controls, demonstrating quantitatively significantly lower tracer uptake in PFA patients in comparison to CBA for SUVmax and FAV with a clear, albeit non-significant lower values of SUVpeak and SUVmean for PFA. All PET parameters after CBA were significantly higher than in controls. PFA patients had significantly higher tracer uptake in comparison to controls for SUVpeak and SUVmean with a trend of higher uptake for SUVmax and FAV

Copyright American Society of Nuclear Cardiology

CONCLUSIONS

1- Tissue response with respect to fibroblast activation seems to be less pronounced in PFA compared to established thermal ablation systems.

2- Fibroblast activation seems to reflect different cell death mechanism and collateral remodeling processes induced by different ablation techniques

3-^{68}Ga-FAPI-PET may help to understand lesion formation after PFA and how it relates to a long-term outcome or possible complications, that at the moment may not be apparent.

4- ^{68}Ga-FAPI-PET may be used as an imaging modality to monitor atrial remodeling in response to tissue damage.

Copyright American Society of Nuclear Cardiology
